# Comparative real-world effectiveness and safety of benralizumab and two mepolizumab dosing regimens in eosinophilic granulomatosis with polyangiitis: a 24-month prospective single-center cohort study

**DOI:** 10.3389/fimmu.2026.1844354

**Published:** 2026-06-15

**Authors:** Marta Codirenzi, Federica Davanzo, Luca Iorio, Eleonora Fiorin, Gabriella Guarnieri, Fulvia Chieco Bianchi, Alessia Achille, Maria Rita Marchi, Andrea Vianello, Andrea Doria, Roberta Ramonda, Roberto Padoan

**Affiliations:** 1 Rheumatology Unit, Department of Medicine (DIMED), University of Padua, Padua, Italy; 2 Respiratory Pathophysiology Division, Department of Cardio-Thoracic-Vascular Sciences, University of Padua, Padua, Italy; 3 Respiratory Unit, Cittadella Hospital, ULSS6 Euganea, Cittadella, Italy

**Keywords:** benralizumab, eosinophilic granulomatosis with polyangiitis, mepolizumab, precision medicine, remission

## Abstract

**Background:**

Long-term prospective real-world data on anti–IL-5 and anti–IL-5 receptor biologics in eosinophilic granulomatosis with polyangiitis (EGPA) are limited.

**Methods:**

In this 24-month prospective single-center observational study, 66 adults with EGPA received benralizumab 30 mg (every 4 weeks for the first three doses, then every 8 weeks), mepolizumab 300 mg every 4 weeks or mepolizumab 100 mg every 4 weeks. Remission was defined as BVASv3 = 0 with prednisone ≤5 mg/day. Glucocorticoid (GC)-free status was defined as no oral GCs. The primary analysis was conducted in patients starting biologics as first-line. To address confounding by indication, we performed multivariable logistic regression and multinomial propensity score inverse probability weighting (IPTW). Analyses were repeated in an all-lines dataset (81 treatment lines) using generalized estimating equations (GEE) clustered by patient.

**Results:**

Clinical outcomes improved over the 24-month follow-up across all regimens. By 24 months, remission was 73.7% (14/19) with benralizumab, 81.0% (17/21) with mepolizumab 300 mg, and 50.0% (6/12) with mepolizumab 100 mg, without statistically significant differences between regimens. GC-free status increased over time, reaching 63.2% (12/19) with benralizumab, 85.7% (18/21) with mepolizumab 300 mg, and 58.3% (7/12) with mepolizumab 100 mg at 24 months, with no significant between-group differences. Adjusted and propensity-based sensitivity analyses yielded consistent conclusions. Eosinophils decreased markedly, with near-complete suppression with benralizumab. Pulmonary function indices showed improvement during follow-up, with the clearest within-group improvement observed in the benralizumab group. Treatment persistence at 24 months varied across regimens, although no statistically significant difference was observed (log-rank p=0.222). Discontinuations were mainly driven by inadequate control of ENT or respiratory manifestations. In all-lines GEE analyses, regimen effects on remission and GC-free status were broadly consistent with the primary analysis. Adverse events were uncommon and no serious events were reported.

**Conclusions:**

Anti–IL-5/IL-5R biologics were effective and well tolerated over 24 months in EGPA, with substantial GC sparing effect. The observational design and potential confounding by indication limit comparative effectiveness inference. These findings support individualized biologic selection in EGPA.

## 1 Introduction

Eosinophilic granulomatosis with polyangiitis (EGPA) is a rare inflammatory disease characterized by asthma, necrotizing vasculitis, extravascular granulomas, and blood and tissue eosinophilia (1). Current treatment recommendations emphasize a severity-driven approach, distinguishing severe disease (organ- or life-threatening involvement and/or higher prognostic risk) from non-severe presentations, and tailoring therapy according to disease phase (new-onset or relapsing disease) (2). In patients with severe disease, induction typically consists of high-dose glucocorticoids (GCs) with cyclophosphamide or rituximab, followed by maintenance strategies that may include conventional disease-modifying antirheumatic drugs (cDMARDs) in combination with GCs.

In non-severe EGPA, GCs alone may induce remission; however, many patients experience early relapses, often involving respiratory manifestations, and remain GC-dependent, resulting in cumulative toxicity (2, 3). Given the central role of eosinophilic inflammation in EGPA, interleukin-5 (IL-5) pathway blockade represents a rational steroid-sparing approach, particularly for patients with relapsing or refractory disease (4, 5). Mepolizumab, a monoclonal antibody targeting IL-5, has demonstrated efficacy and an acceptable safety profile in relapsing and refractory EGPA at a dose of 300 mg every 4 weeks (q4w) (6, 7). In parallel, real-world studies have suggested that mepolizumab 100 mg q4w, approved for severe eosinophilic asthma (8) and chronic rhinosinusitis with nasal polyps (CRSwNP), may also provide clinically meaningful benefit in EGPA, particularly for respiratory symptoms, although comparative data across different dosing regimens remain limited (9, 10).

Benralizumab is a humanized monoclonal antibody targeting the IL-5 receptor α subunit (IL-5Rα), which is expressed on eosinophils, and is approved for the treatment of severe eosinophilic asthma and, more recently, EGPA (11). The phase 3 randomized controlled trial MANDARA demonstrated the non-inferiority of benralizumab 30 mg q4w compared with mepolizumab 300 mg q4w for maintaining remission in relapsing or refractory EGPA patients (11). In severe eosinophilic asthma, benralizumab is administered subcutaneously at a dose of 30 mg q4w for the first three doses, followed by every 8 weeks (q8w) thereafter. Through eosinophil depletion in both blood and tissues (5), benralizumab represents an additional targeted therapeutic option for patients with EGPA and eosinophil-driven disease manifestations.

In real-world EGPA populations characterized by asthma- and ear, nose, and throat (ENT)-predominant disease, benralizumab is often administered according to the dosing schedule approved for severe eosinophilic asthma (12–14), and prospective long-term comparisons with mepolizumab 300 mg and 100 mg regimens are lacking.

Therefore, we evaluated the 24-month effectiveness and safety of three biologic regimens (benralizumab 30 mg administered according to the severe eosinophilic asthma schedule, mepolizumab 300 mg q4w, and mepolizumab 100 mg q4w) in a prospective, single-center cohort of patients with EGPA. The co-primary endpoints were clinical remission and GC-free status, defined as no oral GC use. Secondary endpoints included changes in disease activity and damage, eosinophil counts, asthma and ENT outcomes, treatment persistence, and adverse events.

## 2 Methods

### 2.1 Study design and setting

We conducted a 24-month, prospective, single-center observational cohort study at the Padua Vasculitis Center (Rheumatology Unit, Padua University Hospital), including adult patients with an established clinical diagnosis of EGPA who were treated with anti-IL-5/IL-5R biologics as part of routine clinical care between November 2017 and November 2025. Clinical and laboratory data were collected at biologic initiation (baseline, T0) and at prespecified follow-up visits at 3, 6, 12, and 24 months.

The study was approved by the Ethics Committee of Territoriale Area Centro - Est Veneto (USTS CET-ACEV: 6315/AO/25), and all patients provided written informed consent for participation and the use of their data. The study was conducted in accordance with the principles of the Declaration of Helsinki.

### 2.2 Participants and treatment regimens

Eligible participants were adults (≥18 years) with EGPA, classified according to the 2022 American College of Rheumatology/European Alliance of Associations for Rheumatology (ACR/EULAR) classification criteria (15), who started benralizumab or mepolizumab as part of routine clinical care. Benralizumab was administered according to the dosing schedule approved for severe eosinophilic asthma (30 mg subcutaneously every 4 weeks for the first three doses, followed by every 8 weeks thereafter). Mepolizumab was administered at either 100 mg or 300 mg subcutaneously every 4 weeks.

Exclusion criteria included treatment with other biologic agents, use of immunosuppressants for conditions unrelated to EGPA, and the presence of comorbidities requiring chemotherapy or radiotherapy within 12 months before enrollment.

Because some patients switched biologics during routine clinical care, two analytic populations were prespecified. The primary analysis was conducted in the first-line cohort, including only patients who initiated an anti–IL-5/IL-5R biologic as their first biologic treatment (one observation per patient). A sensitivity analysis was performed including all biologic treatment lines, defined as each initiation of an anti–IL-5/IL-5R biologic; in this analysis, a patient could contribute more than one treatment line.

### 2.3 Treatment indications and background therapy

Indications for anti-IL-5/IL-5R biologics prescription reflected local prescribing practices as well as drug labeling and reimbursement criteria. Benralizumab (30 mg q4w for the first three doses, followed by q8w) and mepolizumab 100 mg (q4w) were generally prescribed for severe eosinophilic asthma and/or CRSwNP, whereas mepolizumab 300 mg (q4w) was preferentially used for systemic and eosinophil-driven disease manifestations, in line with recommendations (10).

Treatment selection was not randomized and reflected routine clinical practice, including disease phenotype, disease severity, organ involvement, prior treatment exposure, drug availability over time, and physician judgment. All patients with nasal symptoms received optimized local therapy, including intranasal corticosteroids (INCS, mometasone furoate 50 µg 2 sprays in each nostril once or 2 times a day or budesonide 100 µg 1 spray in each nostril once a day) and saline nasal irrigation. Similarly, patients with asthma were maintained on optimized inhaled therapy throughout follow-up, consisting of inhaled corticosteroids (ICS), inhaled long-acting β_2_-agonists (LABAs), and long-acting muscarinic antagonists (LAMAs), all of which were continued when previously prescribed and clinically indicated.

Oral GC tapering was guided by treating physician judgment and individualized according to clinical response and patient characteristics.

### 2.4 Data collection

Demographic, clinical, and laboratory data at the time of EGPA diagnosis were collected retrospectively from medical records. In contrast, baseline and follow-up assessments were predefined and prospectively performed from the start of biologic therapy (baseline, T0) and at 3, 6, 12, and 24 months.

Two analytic datasets were generated: a first-line cohort including patients who initiated an anti–IL-5/IL-5R biologic as their first biologic treatment (used for the primary analyses), and an expanded dataset including all biologic treatment lines (used for sensitivity analyses). Collected variables included EGPA manifestations captured by the Birmingham Vasculitis Activity Score version 3 (BVASv3) (16), damage accrual by the Vasculitis Damage Index (VDI), blood eosinophil count, C-reactive protein (CRP), and ANCA status and specificity. Concomitant symptomatic medications (e.g., oral antihistamines, long-acting muscarinic antagonist, long-acting beta-agonist and inhaled corticosteroids) were not systematically recorded. Adverse events occurring during treatment were prospectively recorded at scheduled follow-up visits and verified through medical records at each timepoint. Serious adverse events were defined according to standard regulatory criteria (death, life-threatening event, hospitalization, significant disability/incapacity, or other medically important event) (17).

### 2.5 Respiratory and rhinologic assessment

Pulmonary evaluation included pre-bronchodilator spirometry (forced expiratory volume in 1 second [FEV1, L and % predicted], forced vital capacity [FVC, L and % predicted]) and forced expiratory flow at 25-75% [FEF25-75, L/s and % predicted], all performed according to international standards (18). Fractional exhaled nitric oxide (FeNO, ppb) was measured as a marker of type 2 airway inflammation, and asthma symptom control was assessed by the Asthma Control Test (ACT) (19). Severe asthma and uncontrolled asthma were defined according to European Respiratory Society/American Thoracic Society (ERS/ATS) criteria (8). Severe CRSwNP was defined by a nasal polyp score (NPS) ≥5 with inadequate control despite intranasal corticosteroids, together with either ≥2 systemic GC courses in the prior year or a history of endoscopic sinus surgery; sinonasal quality-of-life (QoL) questionnaires were not systematically collected.

### 2.6 Outcomes

Clinical response was evaluated longitudinally across systemic disease activity, GC exposure, and respiratory and ENT manifestations. Remission and GC-free status were selected as co-primary endpoints and assessed at each follow-up visit (T3, T6, T12 and T24). Remission was defined as BVASv3 of 0 with a daily prednisone-equivalent dose ≤5 mg at the corresponding visit. GC-free status was defined as no oral GC use (daily prednisone-equivalent dose of 0 mg) at the corresponding follow-up visit.

Secondary endpoints included longitudinal changes in BVASv3, VDI, blood eosinophil counts, inflammatory markers (C-reactive protein, CRP), asthma outcomes (exacerbations, ACT score, spirometry parameters, and FeNO), and ENT outcomes.

Asthma exacerbations were defined according to the ERS and European Academy of Allergy and Clinical Immunology (EAACI) criteria (20) and operationalized as episodes requiring systemic GC escalation/burst and/or asthma-related emergency care or acute treatment escalation documented in the clinical record, together with changes in ACT, lung function (FEV1, FVC and FEF25–75), and airway inflammation (FeNO). Uncontrolled ENT disease was based on clinically relevant predefined criteria, specifically the recurrence or worsening of nasal polyps after prior control, the need to initiate systemic glucocorticoid therapy, and/or the requirement for functional endoscopic sinus surgery (FESS), as documented by the treating physicians.

Treatment persistence and discontinuation were assessed as additional outcomes, including the timing of discontinuation, reasons for discontinuation, and subsequent treatment switches. Reasons for discontinuation were classified using predefined categories (inefficacy, adverse events, and other reasons, including planned treatment changes such as dose de-escalation). For longitudinal analyses of the co-primary outcomes, remission and GC-free status were summarized at each visit using available-case denominators. No imputation procedures were applied for missing data. To ensure conservative handling of discontinuations for inadequate disease control, patients who discontinued the index biologic because of insufficient effectiveness were classified as not meeting the corresponding outcome from the time of discontinuation onward. Conversely, patients who discontinued after achieving clinical control (including planned step-down, such as mepolizumab 300 mg to 100 mg) were considered to have achieved the outcome at the time of discontinuation and were subsequently censored in longitudinal analyses.

Treatment failure was classified as primary failure, defined as persistent or recurrent active disease without having achieved remission, and secondary failure, defined as recurrence of disease activity after a previous remission. Adverse events and serious adverse events were prospectively recorded during follow-up and adjudicated from the medical record.

### 2.7 Statistical analysis

Outcomes were compared across the three regimens: benralizumab, mepolizumab 300 mg, and mepolizumab 100 mg. Qualitative variables are presented as absolute numbers and percentages, while continuous variables are reported as median and interquartile range (IQR). The normality of data distribution was assessed using the Shapiro-Wilk test. As the data did not meet the assumptions of normality, non-parametric tests were applied.

Within-group longitudinal changes were assessed using nonparametric methods. For continuous outcomes paired comparisons versus baseline were performed using the Wilcoxon signed-rank test on available paired observations (with multiplicity adjustment for multiple follow-up timepoints where applicable). For binary outcomes, paired changes between two timepoints were assessed using the McNemar test on available paired observations. For repeated binary outcomes across timepoints, within-group changes were assessed using Cochran’s Q test on complete cases. For these exploratory within-group comparisons, a Bonferroni-adjusted significance threshold was applied (p ≤ 0.010). Between-group comparisons at a given timepoint were performed using the Kruskal–Wallis test for continuous variables and the χ² or Fisher’s exact tests for categorical variables, as appropriate. Between-group comparisons at each timepoint were considered exploratory and were interpreted descriptively. To further address potential confounding by indication in this observational study, we performed additional adjusted and propensity-based analyses in the first-line cohort. For the two co-primary endpoints (remission and GC-free status), we fitted multivariable logistic regression models at T24 including treatment regimen (reference: mepolizumab 300 mg) and prespecified baseline covariates: age, sex, disease duration, BVAS at baseline (T0), ANCA status, baseline prednisone-equivalent dose, and baseline eosinophil count. Adjusted effects are reported as adjusted odds ratios (aORs) with 95% confidence intervals (CIs). As a propensity-based sensitivity analysis, we estimated a multinomial propensity score for treatment assignment using the same baseline covariates and applied stabilized inverse probability of treatment weighting (IPTW). IPTW-weighted treatment effects at T24 were estimated using weighted logistic regression with robust standard errors.

Treatment persistence was assessed using Kaplan–Meier methods and compared across regimens using the log-rank test. We further analyzed time to discontinuation using Cox proportional hazards models, reporting hazard ratios (HRs) with 95% CIs. In addition to unadjusted models, adjusted Cox models included the same prespecified baseline covariates listed above.

The primary analyses were performed in the first-line cohort (one observation per patient). Sensitivity analyses were conducted in the dataset including all biologic treatment lines. To account for within-patient correlation arising from repeated treatment lines, we used generalized estimating equations (GEE) with robust (sandwich) standard errors, clustering on patient identifier, including treatment regimen and follow-up timepoints. For binary outcomes (remission and GC-free status), we modeled a binary outcome over time using logistic regression, accounting for correlation within the same patient, and results are reported as odds ratios (ORs) with 95% confidence intervals (CIs). Additional models were used to explore secondary endpoints in sensitivity analyses, including BVAS = 0 (binary), log-transformed eosinophil counts, and log-transformed prednisone-equivalent dose.

All analyses were performed using the free software Jamovi (version 2.7.11) and IBM SPSS Statistics (version 26) (SPSS Inc., Chicago, IL, USA, 2001). Graphics were obtained using GraphPad V10.1.1.

## 3 Results

### 3.1 Disease features at diagnosis and at biologic treatment initiation

A total of 66 patients with EGPA were included in the study, of whom 33 (50.0%) were male. At EGPA diagnosis, the median age was 52 [41.2–58.8] years. Peripheral blood eosinophilia was prominent, with a median eosinophil count of 3,220 [1500–6495] cells/mm³. Twenty-three patients (34.8%) were p-ANCA positive, all with anti-MPO specificity. Clinical involvement at diagnosis was characterized by respiratory manifestations, with asthma in 97.0% and ENT symptoms in 92.4%. Disease activity at diagnosis was moderate to severe, with a median BVAS of 13 [9–18]. Prognostic severity was limited in most patients, with Five Factor Score (FFS) ≥1 in 14/66 (21.2%).

When stratified by the first-line biologic subsequently initiated (benralizumab, mepolizumab 300 mg, or mepolizumab 100 mg), demographic characteristics, ANCA positivity and BVAS at diagnosis were broadly comparable across groups. Clinically relevant differences were observed in prognostic severity and selected organ involvement. FFS ≥1 was more frequent among patients later treated first-line with mepolizumab 300 mg (41.7%) compared with benralizumab (3.8%) and mepolizumab 100 mg (18.8%), p=0.005. Systemic manifestations at diagnosis were more common in the mepolizumab 300 mg group (75.0%) compared with the benralizumab (46.2%) and mepolizumab 100 mg (37.5%) groups, p=0.036. ENT involvement at diagnosis was highly prevalent overall but was less frequent in the mepolizumab 300 mg group (79.2%), p = 0.009. Additional diagnostic features of the cohort are reported in **Table 1**. At biologic initiation (**Table 2**) in the first-line cohort (n=66), median disease duration was 30.5 [4.0–98.5] months, with no evident between-group differences. Systemic activity was generally low-to-moderate (overall median BVAS of 4 [2–8]) and damage (VDI) was similar across regimens. Blood eosinophil counts were elevated overall and showed no clear separation between groups at treatment start. Clinical characteristics at the time of biologic initiation were overall comparable across regimens. The only notable difference was a lower frequency of active asthma at treatment start among patients initiating mepolizumab 300 mg (p=0.016). This finding may be related to timing rather than a true baseline phenotypic difference: patients in the mepolizumab 300 mg group tended to start biologic therapy earlier (although without a statistically significant difference in disease duration), and therefore assessment may have captured the later phase of induction of remission. Prior exposure to immunosuppressive therapy included cyclophosphamide (15.2%), azathioprine (34.8%), methotrexate (34.8%), mycophenolate mofetil (7.6%) and rituximab (15.6%). Previous rituximab use was more common in patients later receiving mepolizumab 300 mg (p=0.005). Concomitant immunosuppressive therapy and glucocorticoid exposure at biologic initiation were broadly comparable across groups, without statistically significant differences.

**Table 1:** Patient demographic and clinical characteristics at EGPA diagnosis.

Characteristic	Overall cohort (N = 66)	Benralizumab (N = 26)	Mepolizumab 100 mg (N = 16)	Mepolizumab 300 mg (N = 24)	P value
Male, n/N (%)	33/66 (50.0%)	13/26 (50.0%)	8/16 (50.0%)	12/24 (50.0%)	1.000
Age, years	52.0 [41.2–58.8]	53.0 [46.0–59.0]	42.0 [37.8–58.0]	54.0 [44.0–58.5]	0.441
ANCA positive, n/N (%)	23/66 (34.8%)	10/26 (38.5%)	2/16 (12.5%)	11/24 (45.8%)	0.084
BVASv3	13 [9–18]	9.5 [7.2–17.5]	13.0 [8.8–18.5]	15.0 [10.8–17.8]	0.137
FFS ≥1, n/N (%)	14/66 (21.2%)	1/26 (3.8%)	3/16 (18.8%)	10/24 (41.7%)	**0.005**
Eosinophils, cells/mm³	3220 [1500–6945]	1965.0 [1512.5–4485.0]	2200 [1320–8050]	5010 [2315–7905]	0.134
Asthma, n/N (%)	64/66 (97.0%)	26/26 (100.0%)	16/16 (100.0%)	22/24 (91.7%)	0.165
ENT involvement, n/N (%)	61/66 (92.4%)	26/26 (100.0%)	16/16 (100.0%)	19/24 (79.2%)	**0.009**
Pulmonary infiltrates, n/N (%)	35/65 (53.8%)	14/26 (53.8%)	9/15 (60.0%)	12/24 (50.0%)	0.831
Peripheral neuropathy, n/N (%)	27/66 (40.9%)	10/26 (38.5%)	7/16 (43.8%)	10/24 (41.7%)	0.940
Extravascular eosinophilic involvement, n/N (%)	24/48 (50.0%)	12/20 (60.0%)	6/12 (50.0%)	6/16 (37.5%)	0.407
Systemic manifestations, n/N (%)	36/66 (54.5%)	12/26 (46.2%)	6/16 (37.5%)	18/24 (75.0%)	**0.036**
Skin involvement, n/N (%)	15/66 (22.7%)	7/26 (26.9%)	1/16 (6.2%)	7/24 (29.2%)	0.192
Cardiac involvement, n/N (%)	12/66 (18.2%)	1/26 (3.8%)	4/16 (25.0%)	7/24 (29.2%)	**0.049**
Gastrointestinal involvement, n/N (%)	2/66 (3.0%)	0/26 (0.0%)	0/16 (0.0%)	2/24 (8.3%)	0.165
Renal involvement, n/N (%)	3/66 (4.5%)	1/26 (3.8%)	0/16 (0.0%)	2/24 (8.3%)	0.453
CNS involvement, n/N (%)	3/66 (4.5%)	0/26 (0.0%)	1/16 (6.2%)	2/24 (8.3%)	0.343
Smoking status, n (%)	Never: 42 (65.6%); Current: 2 (3.1%); Former: 20 (31.2%)	Never: 19 (76.0%); Current: 0 (0.0%); Former: 6 (24.0%)	Never: 14 (87.5%); Current: 0 (0.0%); Former: 2 (12.5%)	Never: 9 (39.1%); Current: 2 (8.7%); Former: 12 (52.2%)	0.011
Body mass index (BMI), kg/m²	24.6 [22.5–26.2]	24.2 [21.8–25.2]	24.7 [24.0–26.3]	24.7 [21.7–26.6]	0.544
Comorbidities, n/N (%)	34/65 (52.3%)	12/26 (46.2%)	9/16 (56.2%)	13/23 (56.5%)	0.720
Occupational exposure to carcinogens, n/N (%)	12/59 (20.3%)	3/22 (13.6%)	3/16 (18.8%)	6/21 (28.6%)	0.469
History of malignancy, n/N (%)	3/64 (4.7%)	0/25 (0.0%)	1/16 (6.2%)	2/23 (8.7%)	0.342

Bold values indicate statistically significant p-values.

**Table 2:** Baseline characteristics at biologic initiation (T0) in the first-line cohort, overall and by biologic regimen.

Characteristic at T0	All cohort (n=66)	Benralizumab (n=26)	Mepolizumab 100 mg (n=16)	Mepolizumab 300 mg (n=24)	p-value
Male, n (%)	33 (50.0%)	13 (50.0%)	8 (50.0%)	12 (50.0%)	1.000
Age, y, median (IQR)	58.0 (47.2–63.0)	57.0 (48.2–63.8)	53.5 (40.5–62.0)	58.5 (51.0–61.5)	0.587
Disease duration, months, median (IQR)	30.5 (4.0–98.5)	39.5 (16.8–98.5)	59.0 (0.0–105.8)	16.5 (3.5–80.5)	0.595
BVASv3, median (IQR)	4.0 (2.0–8.0)	2.0 (2.0–5.0)	4.0 (2.0–6.2)	4.5 (2.0–8.0)	0.229
VDI, median (IQR)	4.0 (2.0–5.0)	3.0 (3.0–5.0)	3.0 (0.0–5.0)	4.0 (2.0–6.0)	0.327
Eosinophils count (/mm3), median (IQR)	890.0 (496.5–1410.0)	850.0 (535.0–1315.0)	705.0 (215.0–1157.5)	1230.0 (496.5–1980.0)	0.403
ANCA-MPO positivity, n (%)	10 (15.2%)	2 (7.7%)	1 (6.2%)	7 (29.2%)	0.056
CRP, mg/L, median (IQR)	2.9 (1.3–5.1)	2.9 (1.8–3.4)	2.9 (1.1–4.5)	2.9 (0.9–5.6)	0.920
FEV1, %, median (IQR)	84.5 (69.8–101.2)	83.0 (65.0–95.2)	82.0 (75.2–88.8)	97.0 (72.5–110.2)	0.355
FVC, %, median (IQR)	93.5 (85.0–105.0)	92.0 (85.8–102.0)	97.5 (83.8–108.2)	98.0 (81.0–112.5)	0.670
FEF 25–75, %, median (IQR)	55.0 (40.0–86.0)	52.0 (24.0–68.0)	46.0 (42.0–85.0)	86.0 (56.0–95.0)	0.242
FeNO, ppb, median (IQR)	41.0 (20.6–56.0)	39.2 (18.7–47.4)	52.0 (29.3–65.2)	32.9 (20.7–48.0)	0.347
ACT, median (IQR)	19.0 (17.0–23.2)	19.0 (16.5–22.5)	20.5 (16.5–22.5)	19.0 (18.0–25.0)	0.875
Clinical manifestations(active), n (%)					
General symptoms	13 (19.7%)	6 (23.1%)	1 (6.2%)	6 (25.0%)	0.295
Pulmonary infiltration	5 (7.6%)	2 (7.7%)	0 (0.0%)	3 (12.5%)	0.342
Asthma	47 (71.2%)	22 (84.6%)	13 (81.2%)	12 (50.0%)	**0.016**
ENT involvement	41 (62.1%)	17 (65.4%)	11 (68.8%)	13 (54.2%)	0.588
Cutaneous involvement	5 (7.6%)	2 (7.7%)	1 (6.2%)	2 (8.3%)	0.970
Cardiac involvement	2 (3.0%)	0 (0.0%)	1 (6.2%)	1 (4.2%)	0.477
Gastrointestinal involvement	2 (3.0%)	0 (0.0%)	0 (0.0%)	2 (8.3%)	0.165
Renal involvement	1 (1.5%)	0 (0.0%)	0 (0.0%)	1 (4.2%)	0.411
Peripheral neuropathy	17 (25.8%)	5 (19.2%)	3 (18.8%)	9 (37.5%)	0.257
Treatments at the time of biologic initiation, n (%)					
Patients on GC, n (%)	52 (78.8%)	23 (88.5%)	10 (62.5%)	19 (79.2%)	0.135
GC, mg/day, median (IQR)	5.0 (2.5–12.5)	7.5 (5.0–12.5)	5.0 (0.0–7.4)	5.0 (2.2–13.8)	0.184
Patients on GC ≥7.5 mg/day, n (%)	28 (42.4%)	13 (50.0%)	4 (25.0%)	11 (45.8%)	0.257
Immunosuppressant, n (%)	21 (31.8%)	6 (23.1%)	5 (31.2%)	10 (41.7%)	0.370
Prior cyclophosphamide, n/N (%)	10/66 (15.2%)	1/26 (3.8%)	3/16 (18.8%)	6/24 (25.0%)	0.102
Prior azathioprine, n/N (%)	23/66 (34.8%)	9/26 (34.6%)	4/16 (25.0%)	10/24 (41.7%)	0.556
Prior methotrexate, n/N (%)	23/66 (34.8%)	13/26 (50.0%)	4/16 (25.0%)	6/24 (25.0%)	0.114
Prior mycophenolate mofetil, n/N (%)	5/66 (7.6%)	1/26 (3.8%)	0/16 (0.0%)	4/24 (16.7%)	0.097
Prior rituximab, n/N (%)	10/64 (15.6%)	2/26 (7.7%)	0/15 (0.0%)	8/23 (34.8%)	0.005
Number of relapses prior to biologic start	0 [0–1]	0 [0–1]	0 [0–1]	0 [0–1]	0.583

Bold values indicate statistically significant p-values.

### 3.2 Co-primary endpoint: clinical remission and glucocorticoid discontinuation

**Figure 1:**
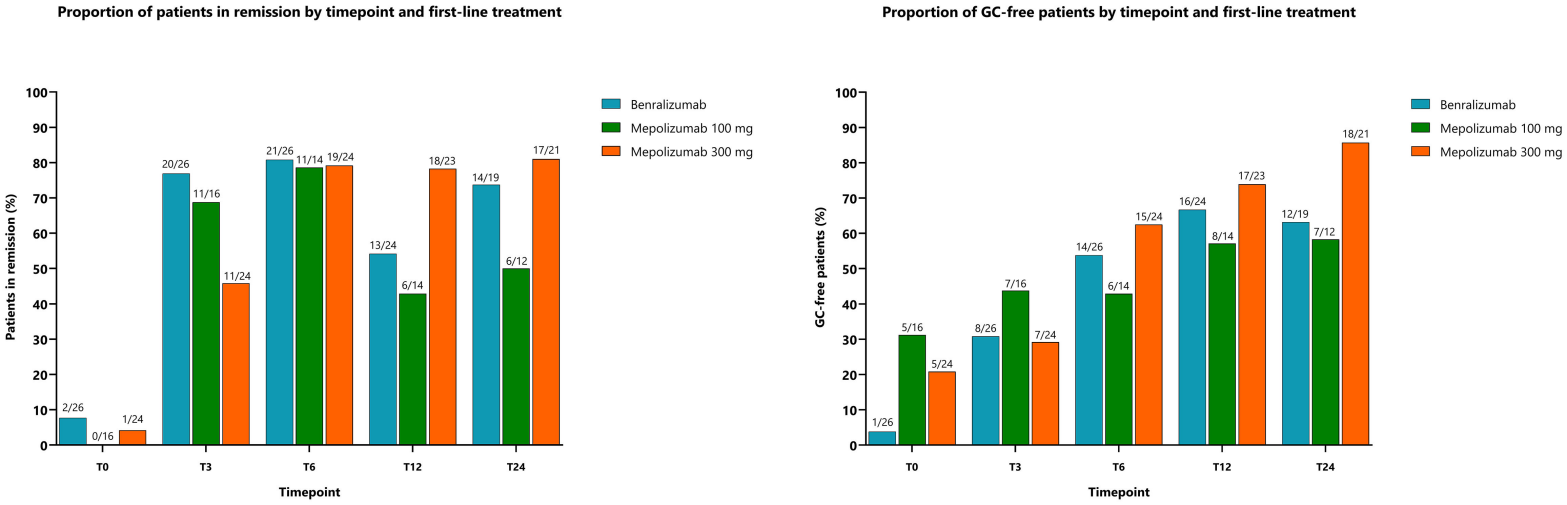
Remission rates and glucocorticoid-free rates following biologics treatment. Remission rates increased progressively over the follow-up period up to 24 months across all three treatment groups (p<0.001 for trend across timepoints). A significant reduction in the proportion of patients receiving glucocorticoids (GC) was observed throughout follow-up in patients treated with benralizumab and mepolizumab 300 mg. Accordingly, the proportion of GC-free patients increased from 1/26 (3.8%) to 12/19 (63.2%) with benralizumab (p<0.001), and from 5/24 (20.8%) to 18/21 (85.7%) with mepolizumab 300 mg (p<0.001). In contrast, the increase observed with mepolizumab 100 mg, from 5/16 (31.2%) to 7/12 (58.3%), did not reach statistical significance (p=0.302).

Overall, remission was achieved in 42/66 (63.6%) patients at T3, 51/64 (79.7%) patients at T6, 37/61 (60.7%) patients at T12 and 37/52 (71.2%) patients at T24. By regimen, remission rates increased over 24 months follow-up from 2/26 (7.7%) to 14/19 (73.7%, p<0.001) with benralizumab, from 1/24 (4.2%) to 17/21 (81.0%, p<0.001) with mepolizumab 300 mg, and from 0/16 (0.0%) to 6/12 (50.0%, p < 0.001) with mepolizumab 100 mg, without statistically significant between-group differences at any timepoints (**Figure 1**, **Supplementary Table 1**).

GC-free status (prednisone dose equal to 0 mg/day) increased progressively over follow-up (p<0.001), rising from 22/66 (33.3%) at T3, to 37/52 (71.2%) at T24 (**Figure 1**). By regimen, GC-free status increased from 2/26 (7.7%) to 12/19 (63.2%, p<0.001) with benralizumab, from 5/24 (20.8%) to 18/21 (85.7%, p<0.001) with mepolizumab 300 mg. In the mepolizumab 100 mg group, GC-free status increased from 6/16 (37.5%) to 7/12 (58.3%), although this within-group change did not reach statistical significance (**Supplementary Table 1**).

Across treatment groups, GC-free rates were comparable at each timepoint (T3 p=0.593; T6 p=0.499; T12 p=0.572; T24 p=0.156). At T24, GC-free status was observed in 12/19 (63.2%) with benralizumab, 18/21 (85.7%) with mepolizumab 300 mg, and 7/12 (58.3%) with mepolizumab 100 mg (**Figure 1**). Beyond GC-free status, daily prednisone dose decreased steadily over follow-up in all groups. Overall, median prednisone-equivalent dose declined from 5.0 [3.1–12.5] mg/day at T0 to 5.0 [0.0–5.9] mg/day at T3, reaching 0.0 [0.0–5.0] mg/day by T6, and remaining low at T12 (0.0 [0.0–4.0] mg/day) and T24 (0.0 [0.0–2.5] mg/day), p<0.001 (T24 vs T0, paired Wilcoxon test). At baseline, median dose was 7.5 [5.0–12.5] mg/day with benralizumab, 5.0 [2.2–13.8] mg/day with mepolizumab 300 mg, and 5.0 [0.0–7.4] mg/day with mepolizumab 100 mg. Median dose fell to 0.0 mg/day by T6 in the benralizumab and mepolizumab 300 mg groups and by T12 in the mepolizumab 100 mg group, with no significant between-group differences at any timepoint. Consistently, the proportion of patients receiving prednisone ≥7.5 mg/day decreased from 28/66 (43.9%) at T0 to 16/66 (24.2%) at T3, 10/64 (15.6%) at T6, 5/61 (8.2%) at T12, and 4/52 (7.7%) at T24, p<0.001 (T24 vs T0, paired McNemar test).

To further address potential confounding by indication, we performed additional adjusted analyses in the first-line cohort. In a multivariable logistic regression model including age, sex, disease duration, baseline BVAS, ANCA status, baseline prednisone-equivalent dose and baseline eosinophil count, no significant between-regimen differences were observed at T24 for either remission or GC-free status. For remission at T24, the adjusted odds ratio (aOR) was 0.45 (95% CI 0.07–2.38; p=0.398) for benralizumab versus mepolizumab 300 mg, and 0.24 (95% CI 0.04–1.92; p=0.188) for mepolizumab 100 mg versus mepolizumab 300 mg; the contrast between benralizumab and mepolizumab 100 mg was also not significant (aOR 1.72, 95% CI 0.28–10.42; p=0.556). Similarly, for GC-free status at T24, no statistically significant differences were observed across regimens (benralizumab vs mepolizumab 300 mg: aOR 0.17, 95% CI 0.02–1.46; p=0.106; mepolizumab 100 mg vs mepolizumab 300 mg: aOR 0.15, 95% CI 0.01–1.51; p=0.107; benralizumab vs mepolizumab 100 mg: aOR 1.16, 95% CI 0.20–6.64; p=0.871).

As a propensity-based sensitivity analysis, we applied IPTW using a multinomial propensity score model based on the same baseline covariates. Weighting improved covariate balance, with the maximum absolute standardized mean difference decreasing from 0.60 before weighting to 0.33 after weighting. In IPTW-weighted analyses, remission at T24 remained comparable across regimens. For GC-free status, however, mepolizumab 100 mg was associated with lower odds of GC discontinuation compared with mepolizumab 300 mg (weighted OR 0.14, 95% CI 0.02–0.83; p=0.030), whereas the other weighted comparisons were not statistically significant. Complete results are reported in **Supplementary Table 2**.

### 3.3 Secondary endpoints: disease activity, damage accrual, eosinophil count and respiratory outcomes

**Figure 2:**
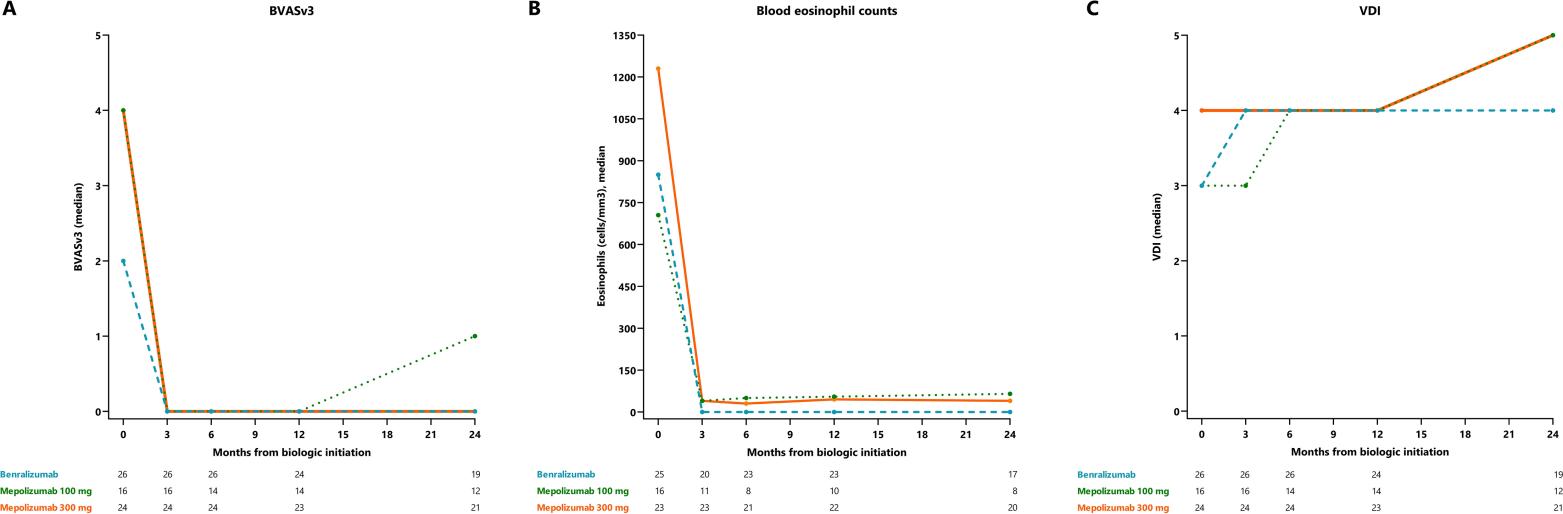
Disease activity, damage accrual, and blood eosinophil counts over follow-up timepoint according to first-line treatment. Light-blue line: benralizumab; green line: mepolizumab 100 mg; orange line: mepolizumab 300 mg. **(A)** A rapid and sustained reduction in BVASv3 was observed in all treatment groups, with near-complete disease control achieved as early as 3 months after treatment initiation (benralizumab p<0.001; mepolizumab 300 mg p<0.001; mepolizumab 100 mg p<0.001) and maintained through follow-up (benralizumab p=0.006; mepolizumab 300 mg p<0.001; mepolizumab 100 mg p=0.006). **(B)** Blood eosinophil counts markedly decreased by 3 months and remained suppressed over time in all groups. Significant differences between groups were observed at each timepoint (between-group p-values: T3 <0.001; T6 p<0.001; T12 p<0.001; T24 p=0.014). **(C)** VDI scores showed small increases over time within each treatment group; however, within-group changes (benralizumab p=0.028; mepolizumab 300 mg p=0.042; mepolizumab 100 mg p=0.016) did not meet the Bonferroni-adjusted threshold for significance (p ≤ 0.010). No significant differences between groups were observed across timepoints.

Disease activity improved rapidly in all treatment groups. At baseline (T0), BVAS was low-to-moderate and did not differ significantly between groups (p=0.361). BVAS decreased significantly by T3 in all groups (Paired T0 vs T3 Wilcoxon p-value: benralizumab p<0.001; mepolizumab 300 mg p<0.001; mepolizumab 100 mg p<0.001) and remained low through follow-up; between-group differences were not significant at T3 (p=0.151) nor at T24 (p=0.158) (**Figure 2**).

Damage accrual over follow-up was limited overall. Baseline VDI did not differ significantly between groups (p=0.295) and between-group differences at T24 were not significant (p=0.326). In paired baseline-to-T24 comparisons, VDI changes were small and did not meet the Bonferroni-adjusted threshold for significance (benralizumab p=0.028; mepolizumab 100 mg p=0.016; mepolizumab 300 mg p=0.042) (**Figure 2**).

Blood eosinophil counts decreased markedly after biologic initiation, with clear between-group separation over follow-up. At T0, eosinophil counts were elevated but comparable across regimens (p=0.399), with medians of 850 [535–1315] cells/mm³ in the benralizumab group, 1230 [496.5–1980] in the mepolizumab 300 mg group, and 705 [215–1157.5] in the mepolizumab 100 mg group. From T3 onward, eosinophil suppression differed significantly across groups (between-group p-values: T3 p<0.001; T6 p<0.001; T12 p<0.001; T24 p=0.014), with near-complete suppression in the benralizumab group and low but detectable levels in the mepolizumab groups (**Figure 2**).

**Figure 3:**
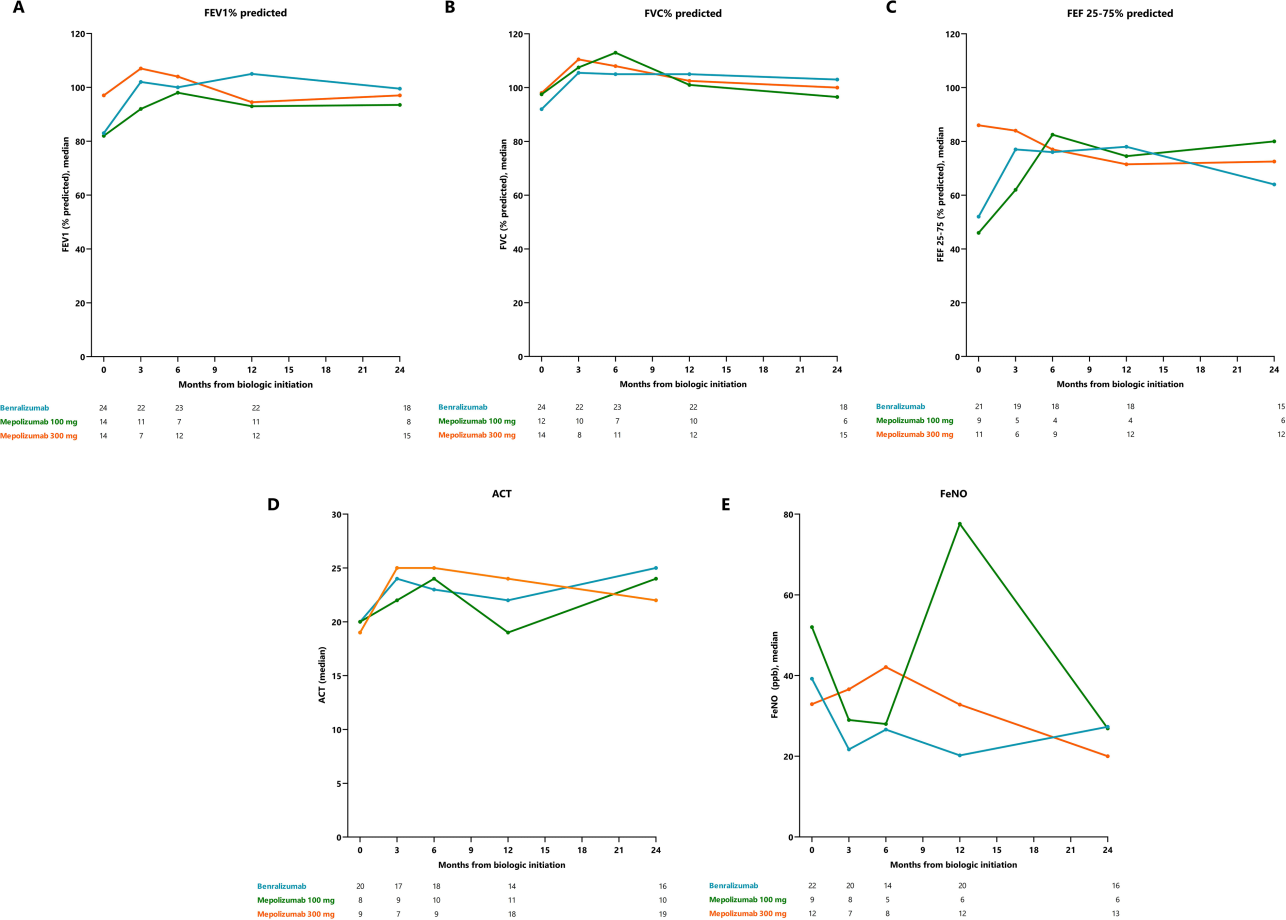
Pulmonary function tests (PFT), FeNO and ACT over follow-up according to first-line treatment. Light-blue line: benralizumab; green line: mepolizumab 100 mg; orange line: mepolizumab 300 mg. **(A–C)** Pulmonary function tests (FEV1% predicted, FVC% predicted, FEF25–75% predicted) improved numerically over time across all treatment groups, but significantly only in the benralizumab group (p<0.001, p=0.001, and p=0.001 respectively). No significant differences between groups were observed at any timepoint. **(D)** ACT scores increased numerically over the follow-up period, without significant between-group differences. **(E)** FeNO levels decreased numerically over time in all treatment groups, with no significant differences at baseline or at T24.

Asthma exacerbations decreased over follow-up in all groups. Overall, the proportion of patients with ≥1 exacerbation fell from 35/66 (53.0%) at baseline (T0) to 7/59 (11.9%) at T24 (p<0.001). By treatment, ≥1 exacerbation decreased from 15/26 (57.7%) to 4/19 (21.1%) in the benralizumab group (p=0.039, not significant after Bonferroni adjustment, paired n=19), from 11/24 (45.8%) to 1/24 (4.2%) in the mepolizumab 300 mg group (p=0.006, paired n=24), and from 9/16 (56.3%) to 2/16 (12.5%) in the mepolizumab 100 mg group (p=0.016, not significant after Bonferroni adjustment, paired n=16). Between-group differences in asthma exacerbation counts were not statistically significant at any timepoint (T0 p=0.677; T3 p=0.080; T6 p=0.875; T12 p=0.445; T24 p=0.240) (**Figure 3**).

Pulmonary function indices improved over time, reaching statistical significance only in the benralizumab group. FEV1% increased significantly from T0 to T24 in benralizumab-treated patients (p<0.001; paired n=18), whereas changes did not reach statistical significance with mepolizumab 300 mg (p=0.672; paired n=9) or mepolizumab 100 mg (p=0.078; paired n=7). FeNO values changed over time across all groups, with no significant between-group differences observed at baseline or at T24. No significant between-group differences were observed for FEV1, FVC and FEF25–75 at any timepoint [FEV1% (T0 p=0.355; T3 p=0.556; T6 p=0.702; T12 p=0.534; T24 p=0.939), FVC% (T0 p=0.670; T3 p=0.984; T6 p=0.905; T12 p=0.829; T24 p=0.760), and FEF25–75% (T0 p=0.242; T3 p=0.935; T6 p=0.905; T12 p=0.892; T24 p=0.828)] (**Figure 3**).

Asthma control improved over follow-up: the median ACT increased from 19.0 [17.0–23.0] at T0 (n=37) to 24.0 [21.0–25.0] at T24 (n=45). In paired analyses, ACT increased in the benralizumab group (T0 19.5 [16.8–22.3] to T24 25.0 [21.0–25.0]; p=0.039; paired n=14), whereas changes in the mepolizumab groups did not reach statistical significance (**Figure 3**).

Complete longitudinal results for secondary endpoints are shown in **Supplementary Table 3**.

### 3.4 Treatment persistence and reasons for discontinuation

Treatment persistence varied across first-line regimens. Overall, 20/66 (30.3%) patients discontinued their first-line biologic during follow-up. Discontinuation was numerically more frequent with mepolizumab 100 mg (7/16, 43.8%) and benralizumab (9/26, 34.6%) than with mepolizumab 300 mg (4/24, 16.7%), although not significant. Among discontinuers, the median time to discontinuation was 13.5 (IQR 6–21) months. Discontinuations occurred predominantly within the first two years: 6/20 (30.0%) occurred within ≤6 months, 4/20 (20.0%) between 6–12 months, and 10/20 (50.0%) between 12–24 months. Median time to discontinuation was 12.0 (IQR 12–17) months with benralizumab, 12.5 (IQR 8.3–17.3) months with mepolizumab 300 mg, and 15.0 (IQR 4.5–23.5) months with mepolizumab 100 mg.

Discontinuations were most commonly driven by inadequate control of ENT manifestations (12/20, 60.0%), followed by persistent asthma/respiratory symptoms (4/20, 20.0%). Less frequent reasons included systemic relapse (1/20, 5.0%), adverse event (1/20, 5.0%), and planned step-down (1/20, 5.0%). Failure-related discontinuations were numerically more frequent with benralizumab (9/26, 34.6%) and mepolizumab 100 mg (6/16, 37.5%) than with mepolizumab 300 mg (3/24, 12.5%), although this difference did not reach statistical significance (p=0.123). By regimen, ENT-driven discontinuations were most frequent with benralizumab (8/9), whereas mepolizumab 100 mg discontinuations reflected a mix of ENT (3/7) and asthma/respiratory (2/7) reasons; mepolizumab 300 mg discontinuations were mainly asthma/respiratory (2/4) or ENT (1/4) with one planned step-down. Among the 20/66 patients who discontinued first-line therapy, 13 (65%) had a subsequent biologic treatment line recorded. In patients who contributed ≥2 biologic treatment lines (n=15), the most frequent first switches were benralizumab to mepolizumab 300 mg (5/15, 33.3%), mepolizumab 100 mg to benralizumab (4/15, 26.7%), mepolizumab 100 mg to mepolizumab 300 mg (3/15, 20.0%), and mepolizumab 300 mg to benralizumab (3/15, 20.0%); overall, switching was most often driven by ENT disease (7/15, 46.7%) or asthma/respiratory activity (4/15, 26.7%). Details on discontinuation reasons and therapy shifts are summarized in **Table 3**, while a swimmer plot illustrating treatment sequencing and duration for patients with ≥2 biologic treatment lines is provided as **Supplementary Figure 1**.

**Table 3:** Discontinuation reasons and therapy switch according to treatment regimen.

Treatment discontinuation/switch variable	Benralizumab 30 mg (q4w×3 then q8w)	Mepolizumab 100 mg/4w	Mepolizumab 300 mg/4w	*p* value
Discontinuation, n, %	9/26 (34.6)	7/16 (43.8)	4/24 (16.7)	0.156
Reason for treatment discontinuation: Failure, n % Primary failure, n % Secondary failure, n % Uncontrolled ENT Uncontrolled asthma Relapse, n % Other, n % Adverse events, n	9/26 (34.6) 2/26 (7.7) 8/26 (30.8) 8/9 (88.9) 0/9 (0.0) 1/26 (3.8) 0/26 (0.0) 0 (0.0)	6/16 (37.5) 2/16 (12.5) 5/16 (31.2) 3/7 (42.9) 2/7 (28.6) 0/16 (0.0) 0/16 (0.0) 1 (6.2)	3/24 (12.5) 1/24 (4.2) 3/24 (12.5) 1/4 (25.0) 2/4 (50.0) 0/24 (0.0) 0/24 (0.0) 0 (0.0)	0.123 0.621 0.243 0.049 0.090 0.458 1.000 0.205
Shift to other biologics, n (%) Benralizumab, n (%) Mepolizumab 100, n (%) Mepolizumab 300, n (%) Dupilumab, n (%)	4/26 (15.4) - 0/26 (0.0) 4/26 (15.4) 0/26 (0.0)	6/16 (37.5) 4/16 (25.0) - 2/16 (12.5) 0/16 (0.0)	3/24 (12.5) 3/24 (12.5) 0/24 (0.0) - 0/24 (0.0)	0.117 0.036 - 0.144 -

**Figure 4:**
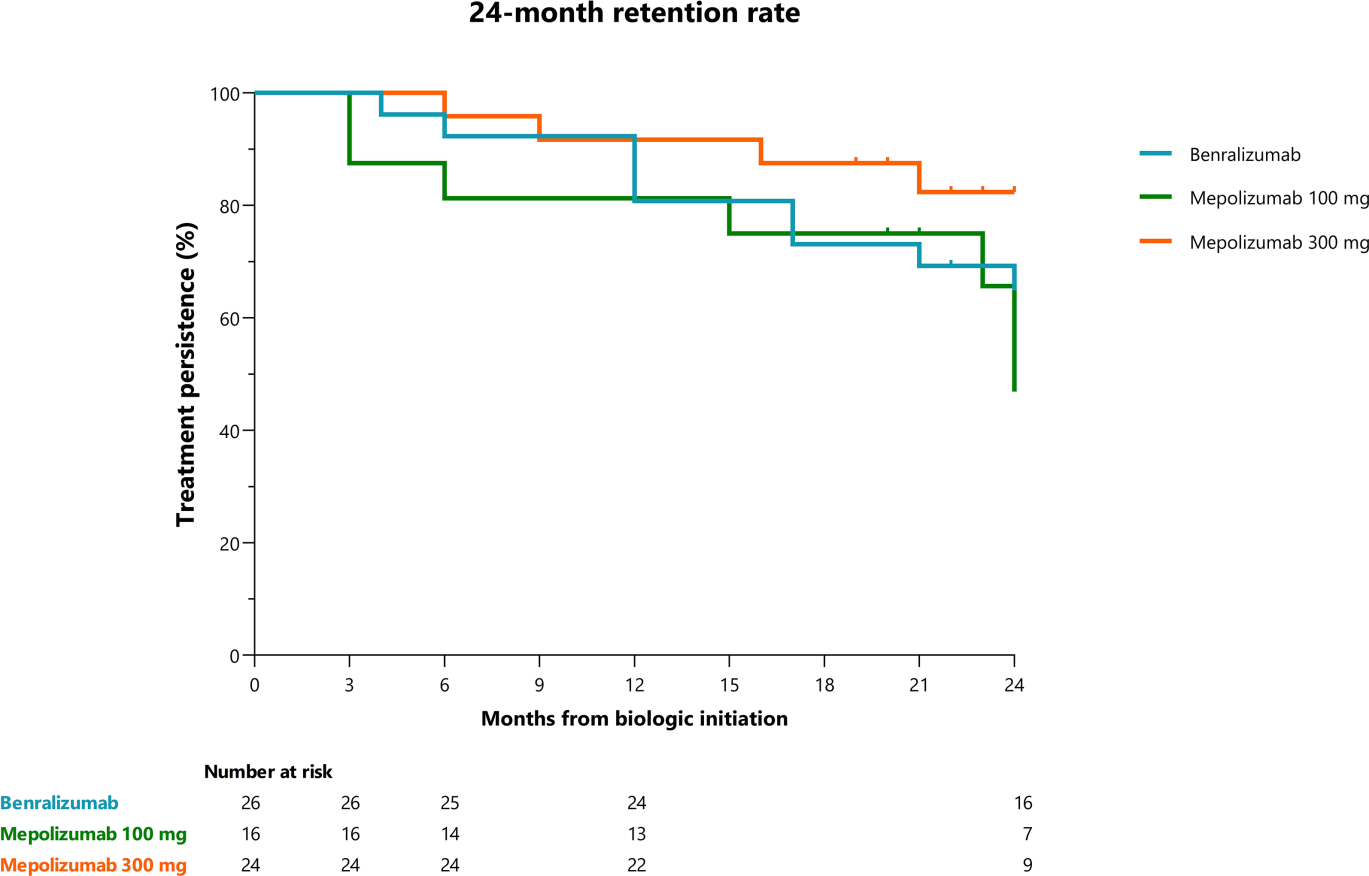
Kaplan–Meier curves showing treatment persistence for patients receiving benralizumab, mepolizumab 100 mg, and mepolizumab 300 mg as first-line biologic therapy. Kaplan–Meier estimates of treatment persistence at 24 months were 64.9% for benralizumab, 82.4% for mepolizumab 300 mg, and 46.9% for mepolizumab 100 mg. Differences between curves were not statistically significant (log-rank p=0.222). Numbers at risk are reported below the x-axis.

Kaplan–Meier estimates of treatment persistence at 12 months were 80.8% for benralizumab, 91.7% for mepolizumab 300 mg, and 81.2% for mepolizumab 100 mg. At 24 months persistence was 64.9%, 82.4%, and 46.9%, respectively. Differences between curves were not statistically significant (log-rank p=0.222) (**Figure 4**).

Finally, we explored treatment persistence using Cox proportional hazards models for time to discontinuation. In the unadjusted model, the hazard of discontinuation did not differ significantly between benralizumab and mepolizumab 300 mg (HR 1.96, 95% CI 0.60–6.38; p=0.265), whereas mepolizumab 100 mg showed a numerically higher, but non-significant, hazard of discontinuation compared with mepolizumab 300 mg (HR 2.84, 95% CI 0.83–9.69; p=0.097). Consistently, the unadjusted benralizumab vs mepolizumab 100 mg contrast was not statistically significant (HR 0.69, 95% CI 0.26–1.86; p=0.463). Similar findings were observed after adjustment for baseline covariates (benralizumab vs mepolizumab 300 mg: adjusted HR 1.85, 95% CI 0.47–7.31; p=0.379; mepolizumab 100 mg vs mepolizumab 300 mg: adjusted HR 3.51, 95% CI 0.82–15.03; p=0.091), with the adjusted benralizumab vs mepolizumab 100 mg contrast remaining non-significant (adjusted HR 0.53, 95% CI 0.18–1.55; p=0.245).

### 3.5 Safety

Adverse events were uncommon and no event requiring hospitalization was recorded at any timepoint. The proportion of patients reporting any adverse events was 6/66 (9.1%) at both T0 and T3, decreasing to 3/62 (4.8%) at T6, 2/60 (3.3%) at T12, and 1/52 (1.9%) at T24. Across regimens, the proportion of patients reporting adverse events remained low throughout follow-up (e.g., at T0: benralizumab 4/26 [15.4%], mepolizumab 300 mg 2/24 [8.3%], mepolizumab 100 mg 0/16 [0%]; at T24: benralizumab 1/19 [5.3%], mepolizumab 300 mg 0/21 [0%], mepolizumab 100 mg 0/12 [0%]). One discontinuation was attributed to an adverse event (ecchymosis) in the mepolizumab 100 mg group.

Adverse events occurred predominantly early after biologic initiation, with the highest frequency within the first 6 months, declining thereafter.

### 3.6 Sensitivity analyses accounting for repeated treatment lines

**Figure 5:**
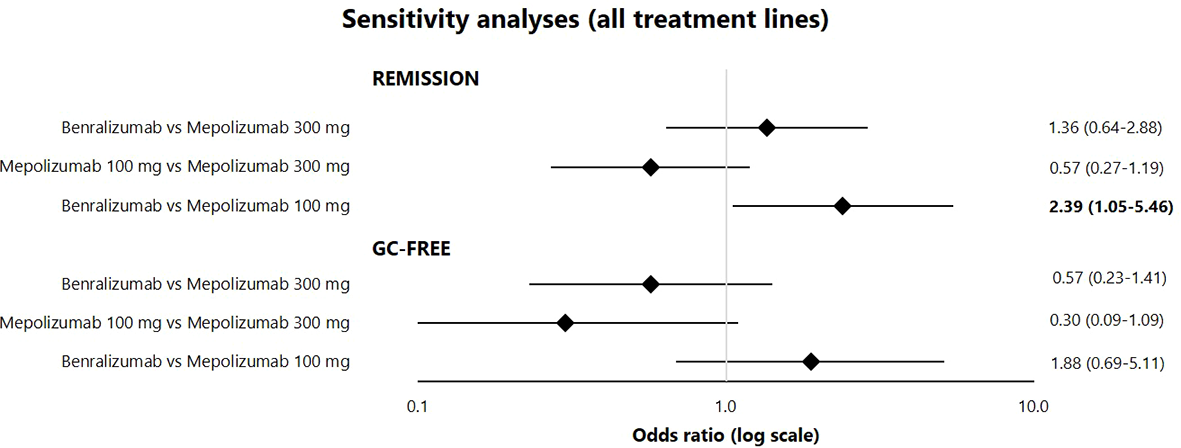
Forest plot showing ORs with 95% CIs for pairwise comparisons between benralizumab, mepolizumab 100 mg, and mepolizumab 300 mg across sensitivity analyses. Estimates are presented on a logarithmic scale, with the vertical line indicating no effect (OR = 1). Models were adjusted for time (T0 as reference) and mepolizumab 300 mg was used as the reference regimen. Using model-based contrasts, benralizumab was associated with higher odds of remission compared with mepolizumab 100 mg (OR 2.39, 95% CI 1.05–5.46; p=0.039). No other comparisons reached statistical significance for remission or GC-free status. OR, odds ratio; CI, confidence interval.

In sensitivity analyses including all biologic treatment lines, we modeled outcomes at each timepoint (T0, T3, T6, T12, T24, with T0 as reference) while accounting for repeated treatment lines within the same patient using GEE with robust (sandwich) standard errors clustered on patient identifier. The all-lines dataset included 81 treatment lines from 66 patients (33 benralizumab, 32 mepolizumab 300 mg, 16 mepolizumab 100 mg), contributing repeated observations across timepoints. After adjustment for timepoint (T0 as reference) and using mepolizumab 300 mg as the reference regimen, treatment effects were broadly consistent with the primary analysis. For remission, benralizumab did not differ significantly from mepolizumab 300 mg (OR 1.36, 95% CI 0.64–2.88; p=0.430), and mepolizumab 100 mg also did not differ significantly from mepolizumab 300 mg (OR 0.57, 95% CI 0.27–1.19; p=0.133); in model-based contrasts, higher odds of remission were observed for benralizumab compared with mepolizumab 100 mg (OR 2.39, 95% CI 1.05–5.46; p=0.039). Similarly, for GC discontinuation (GC-free status), neither benralizumab nor mepolizumab 100 mg differed significantly from mepolizumab 300 mg (benralizumab vs mepolizumab 300 mg: OR 0.57, 95% CI 0.23–1.41; p=0.226; mepolizumab 100 mg vs mepolizumab 300 mg: OR 0.30, 95% CI 0.09–1.09; p=0.068), and the contrast benralizumab vs mepolizumab 100 mg was not significant (OR 1.88, 95% CI 0.69–5.11; p=0.215) (**Figure 5**).

Timepoint effects, referenced to baseline, demonstrated marked improvements over follow-up. The odds of remission were substantially higher at T3 (OR 25.0, 95% CI 11.1–56.4; p<0.001) and peaked at T6 (OR 48.9, 95% CI 19.0–125.9; p<0.001) relative to T0. Similarly, the odds of achieving GC-free status increased progressively over follow-up (T3 vs T0: OR 1.99, 95% CI 1.31–3.01; p=0.001; T24 vs T0: OR 8.04, 95% CI 3.94–16.42; p<0.001) (**Figure 5**).

Eosinophil suppression showed clear regimen differences: compared with mepolizumab 300 mg, benralizumab was associated with a substantially lower eosinophil signal (ratio of geometric means for eosinophils+1: 0.08, 95% CI 0.03–0.22; p<0.001), while mepolizumab 100 mg showed higher eosinophils than mepolizumab 300 mg (1.56, 95% CI 1.02–2.90; p=0.040); benralizumab also showed lower eosinophils than mepolizumab 100 mg (0.03, 95% CI 0.01–0.12; p<0.001). Finally, regarding the prednisone dose, mepolizumab 100 mg was associated with higher values than mepolizumab 300 mg (ratio of geometric means for prednisone+0.5: 2.01, 95% CI 1.02–3.95; p=0.044), whereas benralizumab did not differ significantly from mepolizumab 300 mg (1.36, 95% CI 0.73–2.54; p=0.339).

## 4 Discussion

This prospective single-center study provides real-world data on the 24-month effectiveness and safety of benralizumab and two mepolizumab dosing regimens in patients with EGPA. Overall, all three biologic regimens were associated with improvements in disease activity, respiratory outcomes, and glucocorticoid exposure, with a low frequency of adverse events. Treatment discontinuations were mainly driven by inadequate control of ENT and respiratory manifestations. These findings add prospective long-term real-world evidence in a setting where comparative data across biologic regimens remain limited.

At diagnosis, baseline characteristics were largely comparable across groups, with only few differences that should be interpreted descriptively. By the time of biologic initiation, clinical manifestations were broadly similar across regimens, with the only notable difference being a lower frequency of active asthma in the mepolizumab 300 mg group, possibly reflecting earlier treatment initiation in this subgroup, consistent with the earlier approval of mepolizumab for EGPA and its inclusion in EULAR recommendations (2, 3). Conversely, benralizumab and mepolizumab 100 mg were frequently used in patients with a predominantly eosinophilic phenotype, mainly characterized by severe asthma and ENT involvement, consistent with their mechanism of action and established indications in severe eosinophilic asthma (4, 5, 21).

These findings partially align with the MANDARA trial, which reported comparable remission rates for benralizumab 30 mg q4w and mepolizumab 300 mg q4w at 48 weeks (59% and 56%, respectively) (11). In our cohort, remission rates appeared numerically higher with mepolizumab 300 mg at later timepoints; however, between-regimen differences were not statistically significant and this pattern should be interpreted with caution given differences in treatment allocation and sample size. In addition, a European real-world comparative study reported broadly similar overall effectiveness between benralizumab and mepolizumab, with evidence of deeper eosinophil suppression with benralizumab (22).

Our findings differ from a recent Italian multicenter real-world study, which reported lower remission rates in mepolizumab-treated patients at 12 and 24 months (34.8% and 43.5%, respectively) when compared with benralizumab-treated patients (42.3% and 69.2%, respectively) (23). However, the lack of differentiation between mepolizumab 100 mg and 300 mg in that analysis, together with differences in study populations and treatment allocation, limits direct comparability. In our cohort mepolizumab 100 mg was associated with numerically lower remission rates at later timepoints compared with the other regimens, although between-group differences were not statistically significant and denominators were small. This observation may partly reflect residual confounding and treatment selection in routine care, as mepolizumab 100 mg is often used in patients with predominantly respiratory and/or ENT disease. Previous real-world experience suggests that mepolizumab 100 mg may still be effective in selected EGPA patients, particularly those with milder systemic activity or asthma-predominant manifestations (7, 9).

Glucocorticoid exposure decreased over time across all regimens, supporting the steroid-sparing effect of these biologics. Although between-regimen differences were not statistically significant, mepolizumab 300 mg showed a numerical trend toward earlier glucocorticoid sparing (e.g., higher GC-free rates and fewer patients on higher GC doses at intermediate visits), which may reflect its preferential use in patients with systemic disease and its intended role in systemic disease management (6, 7).

All three regimens were associated with a marked reduction in blood eosinophil counts within the first three months, with benralizumab achieving near-complete depletion, consistent with its mechanism of action (24). Respiratory outcomes improved across all treatment groups over follow-up, supporting the role of anti–IL-5/IL-5R therapies in controlling asthma-related manifestations of EGPA. Within-group improvements were most consistently observed in the benralizumab group; however, between-regimen differences were not statistically significant at individual timepoints, so comparative inferences remain limited. Asthma exacerbations decreased over time across all groups, consistent with the known effect of anti–IL-5/IL-5R therapies in reducing exacerbation burden, although no significant differences between treatments were observed at individual timepoints. Taken together, these findings indicate an overall improvement in respiratory outcomes across regimens, with more consistent improvements in lung function and asthma control observed in the benralizumab group. This may be clinically relevant in patients with respiratory-predominant disease, although the absence of significant between-regimen differences limits comparative interpretation (25, 26).

Overall, treatment discontinuation occurred in 34.6% of patients receiving benralizumab, 16.7% receiving mepolizumab 300 mg, and 43.8% receiving mepolizumab 100 mg, without statistically significant between-group differences. Among discontinuations attributed to lack of efficacy, secondary failure was the predominant pattern across all treatments. Persistent ENT symptoms, particularly CRSwNP, represented the main driver of discontinuation among patients treated with benralizumab, whereas discontinuations in the mepolizumab groups were more commonly related to persistent respiratory or combined respiratory/ENT manifestations. This observation may be influenced by the multifactorial biology of CRSwNP, in which pathways upstream of IL-5 (e.g., IL-4/IL-13) can contribute to disease persistence despite effective eosinophil depletion (27–30). Treatment persistence varied across regimens, with numerically higher rates for mepolizumab 300 mg and lower rates for mepolizumab 100 mg at longer follow-up. However, Kaplan–Meier and Cox analyses did not show statistically significant between-regimen differences, and these findings should be interpreted cautiously given differences in treatment indication, disease phenotype, and reasons for switching.

Adverse events were uncommon, with no serious adverse events requiring hospitalization or leading to treatment discontinuation in our cohort. Their frequency remained low across regimens, consistent with the favorable safety profiles of these biologics (6, 7, 13, 26). However, event types were not systematically captured using a predefined structured classification, therefore under-reporting, limited granularity, and the lack of exposure-adjusted rates cannot be excluded.

In sensitivity analyses accounting for repeated treatment lines, findings were broadly consistent with the primary analysis. Expected differences in eosinophil suppression were observed across regimens, with greater eosinophil depletion in the benralizumab group, while prednisone exposure was lower with mepolizumab 300 mg than with mepolizumab 100 mg, supporting a cautious, exploratory interpretation. Adjusted and propensity-based analyses broadly supported the primary conclusions, although residual confounding cannot be excluded.

Our study presents several strengths. First, its prospective design, predefined outcome measures, and standardized follow-up assessments over 24 months allowed for a longitudinal evaluation of treatment effectiveness and safety. In addition, the multidisciplinary approach, involving pulmonologists, and otorhinolaryngologists, enabled systematic assessment of pulmonary function and clinically relevant ENT outcomes. Moreover, despite other comparative studies existing, few have directly compared all three treatment regimens within the same cohort.

However, several limitations must be acknowledged. First, treatment allocation was not randomized and reflected disease phenotype, prior treatment exposure, and drug availability over time. A possible confounding by indication, together with temporal changes in therapeutic strategies and drug availability over time, limits the validity of head-to-head comparisons between regimens and may have biased comparisons across treatment groups. Second, the sample size, although relatively large for a single-center EGPA cohort, remains modest, especially for subgroup analyses according to biologic line, phenotype, or ANCA status. Some clinically relevant differences may have been missed due to limited statistical power. In addition, given the number of secondary endpoints and timepoint-specific comparisons, and the progressive reduction in denominators over follow-up, our analyses are vulnerable to both type I error and type II error; therefore, between-regimen findings should be interpreted as exploratory/hypothesis-generating rather than definitive. Furthermore, the use of available-case analyses without imputation may have introduced bias if missingness was related to treatment response, discontinuation, or disease severity.

## 5 Conclusion

In this real-world cohort, benralizumab and both mepolizumab dosing regimens were associated with improvements across key endpoints, including remission, respiratory outcomes, and glucocorticoid reduction. Treatment discontinuation rates did not differ significantly across regimens. When discontinuation occurred for lack of efficacy, secondary failure was most common, and persistent ENT/CRSwNP was a frequent driver, particularly among benralizumab-treated patients. All biologics demonstrated a favorable safety profile, with no serious adverse events observed. Overall, these findings support a phenotype-driven approach to biologic selection in EGPA, emphasizing the importance of baseline disease activity, organ involvement and the need for long-term control of respiratory and ENT manifestations.

## Data Availability

The raw data supporting the conclusions of this article will be made available by the authors, without undue reservation.
