# Supplementary material for: Comparative real-world effectiveness and safety of benralizumab and two mepolizumab dosing regimens in eosinophilic granulomatosis with polyangiitis: a 24-month prospective single-center cohort study
Source: Front Immunol. 2026 Jun 15;17:1844354. doi: 10.3389/fimmu.2026.1844354 (PMC13310669; doi:10.3389/fimmu.2026.1844354)
Supplement: Supplementary file 1 [file DataSheet1.docx]

Supplementary Material

# Supplementary Tables

**Supplementary Table 1. Co-primary endpoints by timepoint in the first-line cohort (n=66).**

|  | T0 | T3 | T6 | T12 | T24 | P value (across time-points) |
| --- | --- | --- | --- | --- | --- | --- |
| **Remission, n/N (%)** |  |  |  |  |  |  |
| Benralizumab | 2/26  (7.7%) | 20/26 (76.9%) | 21/26 (80.8%) | 13/24 (54.2%) | 14/19 (73.7%) | **<0.001** |
| Mepolizumab 100 mg | 0/16  (0.0%) | 11/16 (68.8%) | 11/14 (78.6%) | 6/14  (42.9%) | 6/12  (50.0%) | **<0.001** |
| Mepolizumab 300 mg | 1/24  (4.2%) | 11/24 (45.8%) | 19/24 (79.2%) | 18/23 (78.3%) | 17/21 (81.0%) | **<0.001** |
| Overall | 3/66  (4.5%) | 42/66 (63.6%) | 51/64 (79.7%) | 37/61 (60.7%) | 37/52 (71.2%) | **<0.001** |
| P value (between groups) | 0.506 | 0.065 | 0.983 | 0.072 | 0.161 |  |
|  | | | | | | |
| **GC-free status , n/N (%)** |  |  |  |  |  |  |
| Benralizumab | 2/26  (7.7%) | 8/26  (30.8%) | 14/26 (53.8%) | 16/24 (66.7%) | 12/19 (63.2%) | **<0.001** |
| Mepolizumab 100 mg | 6/16  (37.5%) | 7/16  (43.8%) | 6/14  (42.9%) | 8/14  (57.1%) | 7/12  (58.3%) | 0.545 |
| Mepolizumab 300 mg | 5/24  (20.8%) | 7/24  (29.2%) | 15/24 (62.5%) | 17/23 (73.9%) | 18/21 (85.7%) | **<0.001** |
| Overall | 13/66 (19.7%) | 22/66 (33.3%) | 35/64 (54.7%) | 41/61 (67.2%) | 37/52 (71.2%) | **<0.001** |
| P value (between groups) | 0.061 | 0.593 | 0.499 | 0.572 | 0.156 |  |

Values are n/N (%). P values compare the three treatment groups at each timepoint (χ² test). Within-regimen changes across timepoints (T0, T3, T6, T12, T24) were assessed for each treatment group using Cochran’s Q test on complete cases. *GC: glucocorticoid. GC-free status: daily oral GC=0 mg.*

**Supplementary Table 2.** Propensity score IPTW sensitivity (multinomial PS; stabilized weights). Weighted treatment effects are reported as wOR (95% CI).

| Outcome | Comparison | N | wOR (95% CI) | p |
| --- | --- | --- | --- | --- |
| Remission at T24 (IPTW) | Benralizumab vs Mepolizumab 300 mg | 52 | 0.81 (0.21–3.15) | 0.759 |
| Remission at T24 (IPTW) | Mepolizumab 100 mg vs Mepolizumab 300 mg | 52 | 0.44 (0.09–2.11) | 0.307 |
| Remission at T24 (IPTW) | Benralizumab vs Mepolizumab 100 mg (contrast) | 52 | 1.82 (0.38–8.80) | 0.458 |
| GC-free at T24 (IPTW) | Benralizumab vs Mepolizumab 300 mg | 52 | 0.27 (0.05–1.30) | 0.102 |
| GC-free at T24 (IPTW) | Mepolizumab 100 mg vs Mepolizumab 300 mg | 52 | 0.14 (0.02–0.83) | **0.030** |
| GC-free at T24 (IPTW) | Benralizumab vs Mepolizumab 100 mg (contrast) | 52 | 1.88 (0.40–8.96) | 0.426 |

**Supplementary Table 3.** Secondary endpoints over time (T0, T3, T6, T12, T24) in the first-line cohort group (n = 66).

| **Variable** | **Group** | **T0** | **T3** | **T6** | **T12** | **T24** | ***p* (T24 vs T0)** |
| --- | --- | --- | --- | --- | --- | --- | --- |
| **BVASv3** | Benralizumab | 2 (2–6), n=26 | 0 (0–0), n=26 | 0 (0–0), n=26 | 0 (0–2), n=24 | 0 (0–1), n=19 | ***0.006*** |
|  | Mepolizumab 100 mg | 4 (2–6), n=16 | 0 (0–0), n=16 | 0 (0–0), n=14 | 0 (0–4), n=14 | 1 (0–2), n=12 | ***0.006*** |
|  | Mepolizumab 300 mg | 4 (2–8), n=24 | 0 (0–2), n=24 | 0 (0–0), n=24 | 0 (0–0), n=23 | 0 (0–0), n=21 | ***<0.001*** |
|  | *p* (between groups) | *0.361* | *0.151* | *0.989* | *0.149* | *0.158* |  |
|  | | | | | | | |
| **Eosinophils (cells/mm³)** | Benralizumab | 850 (540–1360), n=25 | 0 (0–0), n=20 | 0 (0–0), n=23 | 0 (0–0), n=23 | 0 (0–10), n=17 | ***<0.001*** |
|  | Mepolizumab 100 mg | 705 (215–1158), n=16 | 40 (35–100), n=11 | 50 (30–90), n=8 | 55 (22–92), n=10 | 65 (30–72), n=8 | *0.023* |
|  | Mepolizumab 300 mg | 1230 (496–1980), n=23 | 40 (30–80), n=23 | 30 (10–70), n=21 | 45 (20–78), n=22 | 40 (28–72), n=20 | ***<0.001*** |
|  | *p* (between groups) | *0.399* | ***<0.001*** | ***<0.001*** | ***<0.001*** | ***0.014*** |  |
|  | | | | | | | |
| **CRP (mg/L)** | Benralizumab | 2.9 (1.6–3.2), n=20 | 2.9 (0.9–2.9), n=20 | 2.9 (1.5–3.6), n=24 | 2.8 (0.9–3.3), n=23 | 2.9 (2.4–4.8), n=19 | *0.638* |
|  | Mepolizumab 100 mg | 2.9 (1.1–4.5), n=11 | 1.8 (1.0–2.9), n=9 | 0.6 (0.5–2.9), n=7 | 1.0 (0.6–2.6), n=9 | 0.9 (0.8–2.3), n=8 | *0.844* |
|  | Mepolizumab 300 mg | 2.9 (0.9–5.6), n=23 | 1.5 (0.8–2.9), n=24 | 1.4 (0.6–2.9), n=20 | 1.7 (0.5–2.9), n=21 | 1.0 (0.6–1.9), n=20 | *0.017* |
|  | *p* (between groups) | *0.958* | *0.834* | ***0.058*** | *0.413* | ***0.004*** |  |
|  | | | | | | | |
| **FEV1 (% predicted)** | Benralizumab | 83.0 (65.0–95.2), n=24 | 102.0 (87.0–111.2), n=22 | 100.0 (90.0–111.0), n=23 | 105.0 (87.2–113.0), n=22 | 99.5 (83.0–108.8), n=18 | ***<0.001*** |
|  | Mepolizumab 100 mg | 82.0 (75.2–88.8), n=14 | 92.0 (88.5–102.5), n=11 | 98.0 (85.5–104.5), n=7 | 93.0 (81.0–97.5), n=11 | 93.5 (85.8–104.0), n=8 | *0.078* |
|  | Mepolizumab 300 mg | 97.0 (72.5–110.2), n=14 | 107.0 (88.0–109.5), n=7 | 104.0 (94.0–109.5), n=12 | 94.5 (83.2–111.5), n=12 | 97.0 (85.5–108.0), n=15 | *0.672* |
|  | *p* (between groups) | *0.355* | *0.556* | *0.702* | *0.534* | *0.939* |  |
|  | | | | | | | |
| **FVC (% predicted)** | Benralizumab | 92.0 (85.8–102.0), n=24 | 105.5 (102.0–114.5), n=22 | 105.0 (99.0–117.5), n=23 | 105.0 (96.0–113.8), n=22 | 103.0 (86.8–108.5), n=18 | ***0.001*** |
|  | Mepolizumab 100 mg | 97.5 (83.8–108.2), n=12 | 107.5 (99.8–113.8), n=10 | 113.0 (96.0–116.5), n=7 | 101.0 (99.2–107.8), n=10 | 96.5 (94.2–100.2), n=6 | *0.750* |
|  | Mepolizumab 300 mg | 98.0 (81.0–112.5), n=14 | 110.5 (97.0–112.2), n=8 | 108.0 (103.0–112.0), n=11 | 102.5 (86.5–116.2), n=12 | 100.0 (93.5–108.0), n=15 | *1.000* |
|  | *p* (between groups) | *0.670* | *0.984* | *0.905* | *0.829* | *0.760* |  |
|  | | | | | | | |
| **FEF25–75 (% predicted)** | Benralizumab | 52.0 (24.0–68.0), n=21 | 77.0 (62.0–96.0), n=19 | 76.0 (57.8–103.0), n=18 | 78.0 (58.2–98.8), n=18 | 64.0 (52.0–93.5), n=15 | ***0.001*** |
|  | Mepolizumab 100 mg | 46.0 (42.0–85.0), n=9 | 62.0 (60.0–96.0), n=5 | 82.5 (65.0–100.2), n=4 | 74.5 (59.8–84.8), n=4 | 80.0 (62.2–111.2), n=6 | *0.125* |
|  | Mepolizumab 300 mg | 86.0 (56.0–95.0), n=11 | 84.0 (65.5–88.2), n=6 | 77.0 (70.0–86.0), n=9 | 71.5 (49.0–96.0), n=12 | 72.5 (54.8–112.0), n=12 | *0.812* |
|  | *p* (between groups) | *0.242* | *0.935* | *0.905* | *0.892* | *0.828* |  |
|  | | | | | | | |
| **FeNO (ppb)** | Benralizumab | 39.2 (18.7–47.4), n=22 | 21.7 (16.8–34.5), n=20 | 26.6 (14.8–41.5), n=14 | 20.2 (13.0–30.5), n=20 | 27.3 (16.8–40.0), n=16 | *0.389* |
|  | Mepolizumab 100 mg | 52.0 (29.3–65.2), n=9 | 29.0 (23.0–87.2), n=8 | 28.0 (25.9–72.0), n=5 | 77.6 (21.7–209.2), n=6 | 26.9 (21.3–40.9), n=6 | *0.125* |
|  | Mepolizumab 300 mg | 32.9 (20.7–48.0), n=12 | 36.6 (12.6–40.6), n=7 | 42.1 (17.5–91.0), n=8 | 32.8 (22.0–48.5), n=12 | 20.0 (11.1–48.9), n=13 | *0.461* |
|  | *p* (between groups) | *0.347* | *0.520* | *0.537* | *0.110* | *0.704* |  |
|  | | | | | | | |
| **ACT** | Benralizumab | 20 (17–22), n=20 | 24 (22–25), n=17 | 23 (20–25), n=18 | 22 (20–25), n=14 | 25 (21–25), n=16 | *0.039* |
|  | Mepolizumab 100 mg | 20 (16–22), n=8 | 22 (20–25), n=9 | 24 (22–25), n=10 | 19 (18–24), n=11 | 24 (21–25), n=10 | *0.438* |
|  | Mepolizumab 300 mg | 19 (18–25), n=9 | 25 (24–25), n=7 | 25 (23–25), n=9 | 24 (21–25), n=18 | 22 (20–24), n=19 | *0.773* |
|  | *p* (between groups) | *0.898* | *0.147* | *0.261* | *0.199* | ***0.039*** |  |
|  | | | | | | | |
| **VDI** | Benralizumab | 3 (3–5), n=26 | 4 (3–5), n=26 | 4 (3–5), n=26 | 4 (3–5), n=24 | 4 (3–5), n=19 | *0.028* |
|  | Mepolizumab 100 mg | 3 (0–5), n=16 | 3 (2–5), n=16 | 4 (2–6), n=14 | 4 (3–6), n=14 | 5 (3–6), n=12 | *0.016* |
|  | Mepolizumab 300 mg | 4 (2–6), n=24 | 4 (3–6), n=24 | 4 (3–6), n=24 | 4 (3–6), n=23 | 5 (4–6), n=21 | *0.042* |
|  | *p* (between groups) | *0.295* | *0.284* | *0.837* | *0.910* | *0.326* |  |
|  | | | | | | | |

Values are reported as median (IQR), with available n. Between-group comparisons at each timepoint p values are Kruskal–Wallis tests and are presented as exploratory/descriptive (not multiplicity-adjusted). Within-group changes refer to paired comparisons between T24 and baseline (T0) and were evaluated with Wilcoxon signed-rank tests on paired available data; for these exploratory within-group comparisons, a Bonferroni-adjusted significance threshold was applied (p≤0.010) and values meeting this threshold are shown in bold. *ACT: asthma control test; BVASv3: Birmingham Vasculitis Activity Score version 3; CRP: C-reactive protein; FEV1: forced expiratory volume in the first second; FeNO: Fractional exhaled nitric oxide; FVC: forced vital capacity; FEF 25-75: Forced Expiratory Flow at 25–75; VDI: Vasculitis Damage Index.*

*
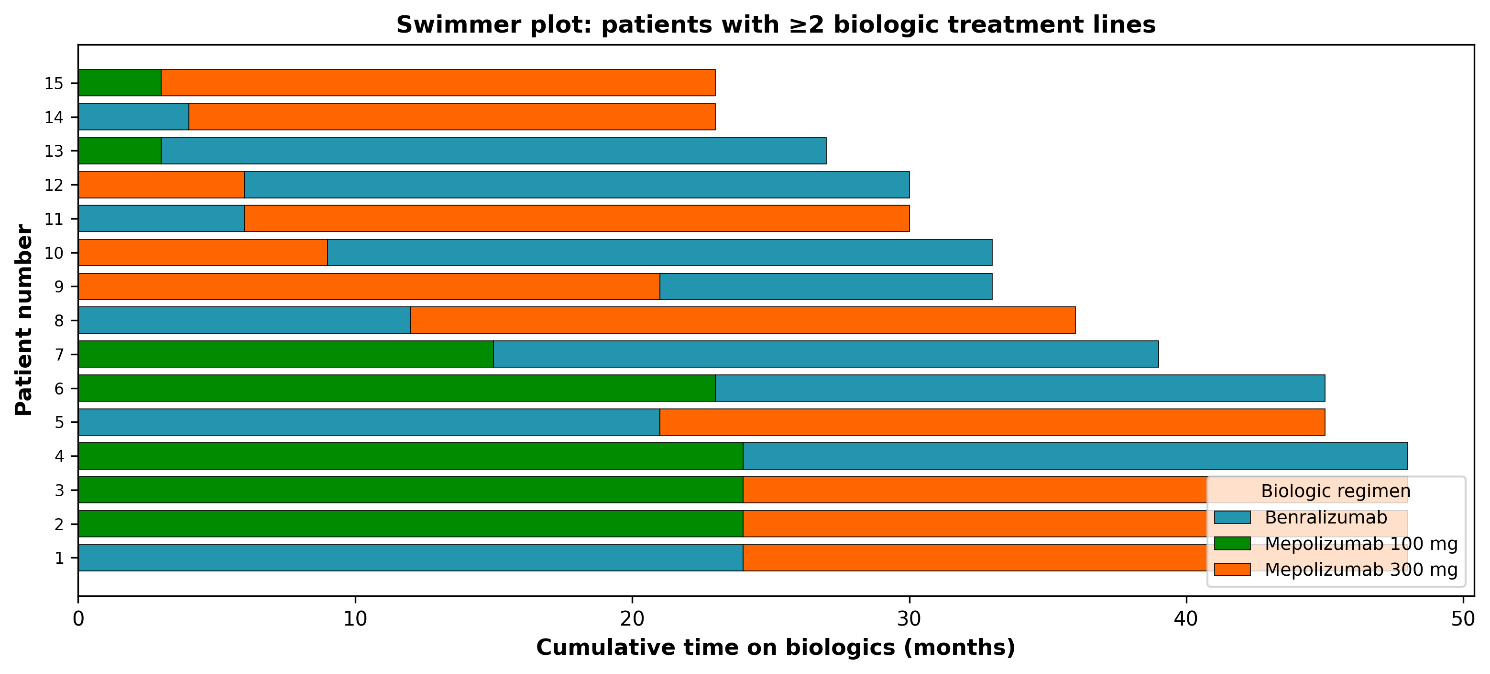
*

**Supplementary Figure 1. Swimmer plot of biologic treatment sequences in switchers**. Swimmer plot depicting the sequence and duration of anti–IL-5/IL-5R biologic treatment lines among patients who received ≥2 biologic treatment lines during follow-up. Each horizontal bar represents one patient (shown as a progressive patient number on the y-axis), and colored segments indicate consecutive treatment lines; segment length corresponds to the time on that regimen (months). The x-axis shows cumulative time on biologic therapy from the start of the first biologic line.
